# Critical State of Sand Matrix Soils

**DOI:** 10.1155/2014/290207

**Published:** 2014-03-16

**Authors:** Aminaton Marto, Choy Soon Tan, Ahmad Mahir Makhtar, Tiong Kung Leong

**Affiliations:** ^1^Faculty of Civil Engineering, Construction Research Centre, Universiti Teknologi Malaysia (UTM), 81310 Johor Bahru, Johor, Malaysia; ^2^Faculty of Management and Human Resource Development, Universiti Teknologi Malaysia (UTM), 81310 Johor Bahru, Johor, Malaysia

## Abstract

The Critical State Soil Mechanic (CSSM) is a globally recognised framework while the critical states for sand and clay are both well established. Nevertheless, the development of the critical state of sand matrix soils is lacking. This paper discusses the development of critical state lines and corresponding critical state parameters for the investigated material, sand matrix soils using sand-kaolin mixtures. The output of this paper can be used as an interpretation framework for the research on liquefaction susceptibility of sand matrix soils in the future. The strain controlled triaxial test apparatus was used to provide the monotonic loading onto the reconstituted soil specimens. All tested soils were subjected to isotropic consolidation and sheared under undrained condition until critical state was ascertain. Based on the results of 32 test specimens, the critical state lines for eight different sand matrix soils were developed together with the corresponding values of critical state parameters, *M*, *λ*, and Γ. The range of the value of *M*, *λ*, and Γ is 0.803–0.998, 0.144–0.248, and 1.727–2.279, respectively. These values are comparable to the critical state parameters of river sand and kaolin clay. However, the relationship between fines percentages and these critical state parameters is too scattered to be correlated.

## 1. Introduction

Recent field evidences of ground failure in sand with limiting percentages of fines during strong earthquakes have highlighted the need to better characterize the stress-strain behaviour of saturated soils in a broader range, from pure sand to sand matrix soils. Although the recent study trend focuses more on the investigation of sand with limiting percentages of fines, the situation is more worsening when there is still no clear conclusion that could be drawn at this moment to describe the roles of fines in liquefaction susceptibility of sand matrix soils. In the absence of a fundamental understanding of the seismic behaviour of sand matrix soils, the usability of the currently used liquefaction susceptibility assessment criteria, the Modified Chinese Criteria that solely relies on the interpretation of few earthquake events, is actually questionable [1–3].

The shear strength and the deformation behaviour of soil are so depended to the combination of changes in volume and confining stress. But the research approach in geotechnical field is to lump together those related postreconnaissance data to formulate new empirical assessment criteria, without capturing their true characteristic through fundamental soil mechanics interpretation. Without implicitly considering the fundamental basis of soil mechanics, applicability of these empirical guidelines which is nonuniversally applicable is arguable. These empirical data only provide limited insight to existing state of art.

The stress-strain behaviour of soils could be either stimulated through constitute models or interpreted within classical plasticity models such as Mohr-Coulomb. Among these models, Critical State Soil Mechanic (CSSM) developed by Schofield and Wroth [4] is the most robust framework to explain the fundamental behaviour of different soil materials. The fact that a loose soil is compressible while a dense soil is dilatants is in general agreement. Density well presented the soil behaviour especially for granular soils. Therefore, CSSM is a powerful tool able to explain the behaviour of soil at various density states. It is a globally recognised framework that the critical states for sand and clay are both well established. Nevertheless, the development of the critical state of sand matrix soils is lacking.

Moreover, CSSM also rooted the basic theoretical framework of soil liquefaction, yet most available findings within the current literature were outside this critical state context.  Table 1 summarizes the overall developments of critical CSSM with respect to soil liquefaction study. This paper aims to discuss the development of the critical state line and corresponded critical state parameters for the investigated material, sand matrix soils using sand-kaolin mixtures. The output of this paper will be used as an interpretation framework for the research of liquefaction susceptibility of sand matrix soils in future.

## 2. The Critical State of Sand

**Figure 1:**
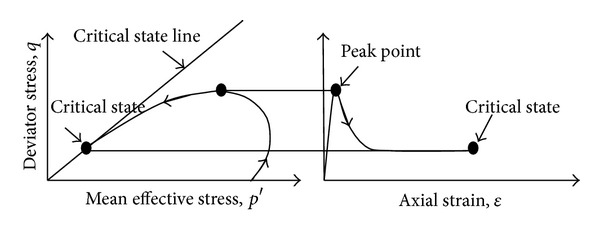
Idealised behaviour of loose particular soil in undrained triaxial shear [10].

To date, quite a number of granular soils have been tested to establish for their critical state.  Figure 1 shows the undrained behaviour of loose sand under triaxial testing. The steady state is exactly representing the critical void ratio under CSSM interpretation. The critical state line (CSL) shows the unique relationship between the deviator stress (*q*), the mean normal effective stress (*p*′), and the specific volume (*ν*). The relationship is as follows:

(1) $$\begin{array}{c} q_{f}=Mp_{f}^{\prime }\\ \nu_{f}=\Gamma -\lambda \ln p_{f}^{\prime } \end{array}$$

in which the subscripts “*f*” denote the ultimate failure at the critical states; *M* denote the critical stress ratio; *λ* denote the gradient of the critical state line; Γ denote the intercept of the critical state line. The parameters of *M*, *λ*, and Γ are regarded as constants for a particular soil and the values for some typical soils are given in Table 2.

Jefferies and Been [5] had summarised some of the important results as shown in Table 3. In fact, the critical state line is not always in its linear relationship especially at stresses higher than 1000 kPa. However, the range of interest in engineering community is lower than 500 kPa; it is true to treat the critical state line in linear form.

Several researchers try to correlate index properties of granular soils with critical state parameter including fines content [6], void ratio [7], and liquidity index [8]. However, findings show that these index properties on their own only give scatter relationship to be correlated with critical state parameters. These findings therefore are too irrelevant to be practically used. However the intrinsic properties of sand including grain size distribution do actually show their significance influence to the critical state line. Clean sand with rounded grains would have a lower value of *λ* (*λ* about 0.03) compared to silty sands with angular shape (*λ* about 0.2).

## 3. Experiment Testing

**Figure 2:**
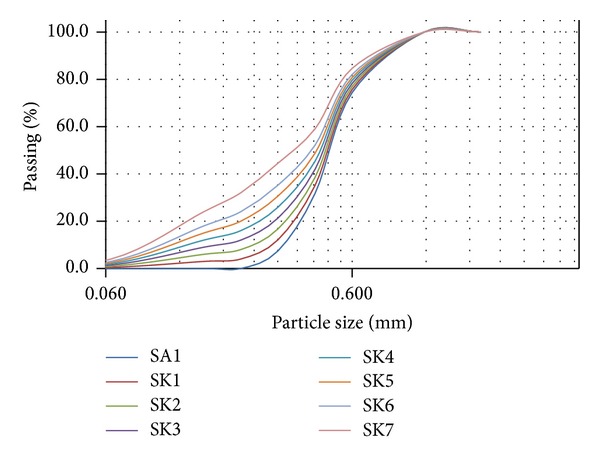
Particle size distribution of the tested soils.

In order to establish the critical state line and the critical state parameters for the investigated material, monotonic triaxial compression tests have been performed. The discussion in this paper based on a total of 32 triaxial tests was carried out on sand-kaolin mixtures at several percentages by weight. The parent sand is uniformly graded medium sand (SP) with specific gravity of 2.63. It was obtained from a river in Johor Bahru, Malaysia. In order to obtain clean sand, it was first rinsed with water to remove impurities before proceeding with the sieve analysis. White kaolin with a specific gravity of 2.62, plastic limit of 38, and liquid limit of 25, manufactured by Kaolin (Malaysia) Sdn Bhd, were added to parent sand to create sand matrix soils with various fines percentages by weight. The index properties of the tested sand matrix soils are presented in Table 4. The specific gravity of all sand matrix soils is therefore also as 2.63. Based on the criteria of coefficient of uniformity (Cu) and coefficient of curvature (Cc) in Unified Soil Classification System, all of the sand matrix soils are classified under uniformly graded soils (SP). Particle size distribution is shown in Figure 2.

The testing conditions were set constantly to increase the precision. All tested specimens have an approximately 100 mm height by 50 mm diameter cylindrical size. Monotonic triaxial compression tests were carried out on strain controlled triaxial apparatus under undrained condition. The specimens were prepared under dry deposition methods to a relative density of 50%. The mould was gently tapped to densify the sand to the required void ratio. The specimens were saturated by being initially flushed with deaired water and followed by increasing the back pressure of 100 kPa. An effective stress of approximately 10 kPa was maintained on the specimen during back pressure saturation. To enhance the consistency of the testing condition, the test was terminated if the specimen could not reach a *B* value of at least 0.96 at this stage. The strain rate of the shearing process is 0.2 mm/min. The specimens were then isotropically consolidated at various effective confining stresses of 50 kPa, 100 kpa, 200 kPa, and 400 kPa. The moisture content was measured at the end of monotonic tests to enable the specific volume at particular mean normal effective stress to be backed calculated.

## 4. Results and Discussion

**Figure 3:**
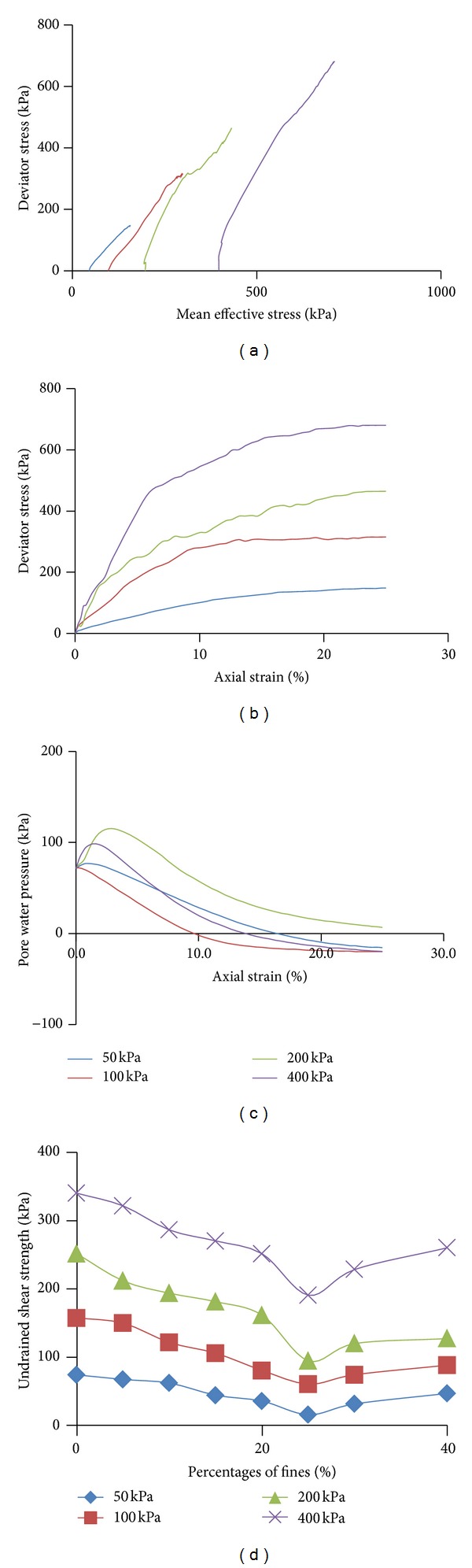
(a) Stress path; (b) peak deviator stress; (c) pore pressure developments; (d) undrained shear strength of tested sand matrix soils.

Figure 3(a) shows the typical stress path of the tested soils performed in this study, particularly the clean sand specimen (SA1). The peak deviator stress is correspondingly increasing at higher effective confining stress as shown in Figure 3(b). It can be observed in Figure 3(c) that the dense specimens (at relative density of 50%) develop negative pore pressure and thus increase the final shear strength. In fact, most researchers prefer to use loose state specimen in establishing the critical state line because of the noticeable peak strength and residue state. However, the existence of quasi-steady state is so confusing and may lead to conservative conclusion. Hence, a dense specimen was considered in this study.  Figure 3(d) shows the peak deviator stress of all 32 monotonic undrained triaxial testings. The hyperbolic curve and the noticeable drop at 25% of kaolin added in sand have been justified in companion paper [9]. The additional of fines will initially facilitate grain separation. At the point of threshold fines content where the fines already fully occupy the interstitial space between the sand grains, it forces a return of the sand matrix soils to far less compressible behaviour.

**Figure 4:**
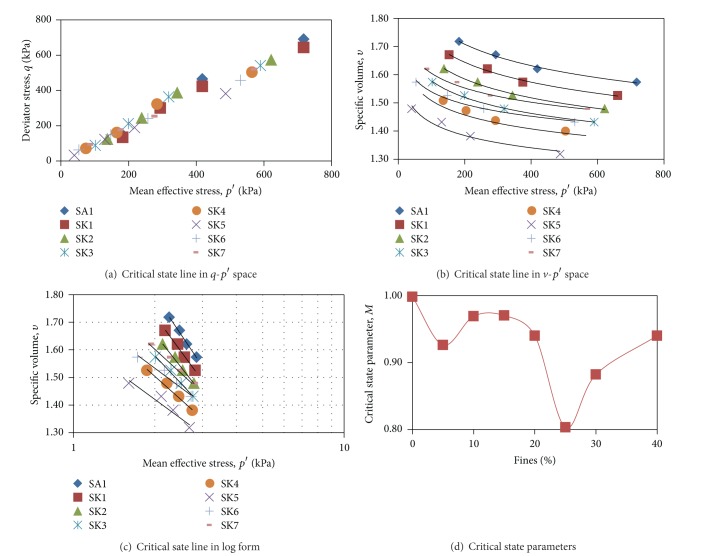
Critical state line and critical state parameters of tested sand matrix soils.

Based on the results of monotonic undrained triaxial testing, the critical state lines of 8 different sand matrix soils are plotted in two different spaces. These critical state lines are parallel to one another. In fact, the straight line through the origin in Figure 4(a) (*q*-*p*′ space) and the curved line in Figure 4(b) (*v*-*p*′ space) are corresponded, when transforming the *v*-*p*′ space into log form as in Figure 4(c); the values of Γ and *λ* could be obtained.  Table 5 summarised the critical state parameters of the tested soils in this study. The range of the value of *M*, *λ*, and Γ is 0.803–0.998, 0.144–0.248, and 1.727–2.279, respectively. These values are comparable to the critical state parameters of river sand and kaolin clay as shown in Table 2. To compare these critical state parameters at different percentages of fines, for example, Figure 4(d) was plotted, particularly the value of *M* across fines percentages. However, there is not an apparent relationship that could be drawn between fines percentages and these critical state parameters. The relationship between fines percentages and these critical state parameters are too scattered to be correlated. In addition, other compositional characteristics including both coefficient of curvature and uniformity, limiting void ratio, and void ratio range are also able to uniquely describe the behaviour of sand matrix soils of different fines percentages. The general indexes that have been usually used to describe the compositional characteristics are not sufficient enough to give good correlations in quantifying the trends of critical state parameters across different percentages of fines added in parent sand. Loosely speaking, the plastic behaviour due to the presence of kaolin, as the plastic fines, is a very potential cause for such variation. The plasticity of higher percentages of kaolin existing within parent sand should have higher values compared to lower percentages. Although it is important to find out which intrinsic factor is actually corresponding to such changes, it is beyond the aims and scope of this paper. Therefore, more researches are warranted in future.

## 5. Conclusion

An experimental study with undrained monotonic triaxial compression test has been conducted on sand-kaolin mixtures (sand matrix soils) and the results were used to establish the critical state of the soils together with corresponding critical state parameters. Based on the results, the following conclusions are achieved.

1. The range of the value of *M*, *λ*, and Γ is 0.803–0.998, 0.144–0.248, and 1.727–2.279, respectively.
2. Neither the fines percentages nor other corresponding compositional characteristics are adequate to be correlated with the critical state parameters of sand matrix soils.
3. More researches are warranted in future to find out which intrinsic factor significantly contributes to the changes to the critical state parameters of sand matrix soils across different fines percentages.

## Figures and Tables

**Table 1:** Overall developments of CSSM with respect to soil liquefaction.

Year	Researchers	Developments
1940	Casagrande [11]	Introduced critical void ratio, the same void ratio where contracted loose soil and dilated dense soil approach after sheared to large strains
1956	Taylor [12]	Showed experimentally that dilatancy is stress dependent
1958	Roscoe et al. [13]	Defined critical state as the ultimate state at which a soil continues to deform at constant stress and constant void ratio
1968	Schofield and Wroth [4]	Brought together stress-dependent strength and dilatancy to introduce critical state soil mechanics with Cam-Clay model
1969	Castro [14]	Observed three different types of stress-strain behaviour (liquefaction, limited liquefaction, and dilation) in monotonic loading tests
1975	Casagrande [15]	Developed steady state line from both drained and undrained tests and noticed that dense sand can be liquefying under sufficient high load
1981	Poulos [16]	Formalised the concept of steady state of deformation (continually deformation under four constant conditions: volume, normal effective stress, shear stress, and velocity)
1985	Poulos et al. [17]	Recognised that steady-state line is useful for identifying the susceptibility of flow liquefaction
1985	Been and Jefferies [18]	Proposed state parameter, the void ratio difference between current state and critical state at same mean stress
1991	Been et al. [19]	Showed that critical state and steady state of sands are equivalent and independent of stress path, sample preparation method, and initial density

**Table 2:** Critical state parameters of some soil types [20].

Soil Indexes	LL	PL	*λ*	Γ	*N*	*M*	*ϕ*′	*κ*/*λ*
Fine-grained clay soils								
London clay	75	30	0.16	2.45	2.68	0.89	23°	0.39
Kaolin clay	65	15	0.19	3.14	3.26	1.00	25°	0.26
Glacial till	35	17	0.09	1.81	1.98	1.18	29°	0.16
Coarse-grained soils								
River sand			0.16	2.99	3.17	1.28	32°	0.09
Decomposed granite			0.09	2.04	2.17	1.59	39°	0.06
Carbonate sand			0.34	4.35	4.80	1.65	40°	0.01

**Table 3:** Critical state properties of some soils (after Jefferies and Been [5]).

Soils	Fines (%)	*e*_max⁡_	*e*_min⁡_	Γ	*λ*_10_	*M*
Castro sand B	0	0.840	0.500	0.791	0.041	1.22
Castro sand C	0	0.990	0.660	0.988	0.038	1.37
Monterey	0	0.820	0.540	0.878	0.029	1.29
Nevada	7.5	0.887	0.511	0.910	0.045	1.2
Ottawa	0	0.790	0.490	0.754	0.028	1.13
Toyoura	0	0.873	0.656	1.000	0.039	1.24
Erksak 330/0.7	0.7	0.747	0.521	0.816	0.031	1.27
Erksak 320/1	1	0.808	0.614	0.875	0.043	1.27
Erksak 355/3	3	0.963	0.525	0.848	0.054	1.18
Chek Lap Kok	0.5	0.682	0.411	0.905	0.13	—

**Table 4:** Compositional characteristic of tested sand matrix soils.

Tested soils	Weight percentages (%)		Density (Mg m^−3^)		Void ratio		Grading	
	Sand	kaolin	Min	Max	Min	Max	Cu	Cc
SA1	100	0	1.37	1.59	0.920	0.649	4.1	1.5
SK1	95	5	1.39	1.66	0.894	0.582	4.8	1.7
SK2	90	10	1.41	1.70	0.867	0.550	5.0	1.2
SK3	85	15	1.43	1.76	0.841	0.491	5.4	1.2
SK4	80	20	1.45	1.80	0.815	0.462	5.7	0.8
SK5	75	25	1.47	1.87	0.788	0.409	5.7	0.4
SK6	70	30	1.39	1.76	0.894	0.491	5.4	0.3
SK7	60	40	1.28	1.63	1.051	0.615	4.6	0.3

**Table 5:** The critical state parameters.

Soils	*M*	Γ	*λ*
SA1	0.998	2.279	0.248
SK1	0.926	2.180	0.232
SK2	0.969	2.094	0.221
SK3	0.970	1.956	0.189
SK4	0.940	1.840	0.166
SK5	0.803	1.727	0.148
SK6	0.882	1.829	0.144
SK7	0.940	1.944	0.269
